# Increased Oxidative Stress and Decreased Sirtuin-3 and FOXO3 Expression Following Carotid Artery Intimal Injury in Hyperlipidemic Yucatan Microswine

**DOI:** 10.26502/fccm.92920355

**Published:** 2024-01-22

**Authors:** Prathosh Velpuri, Parth Patel, Armand Yazdani, Arian Abdi, Vikrant Rai, Devendra K. Agrawal

**Affiliations:** Department of Translational Research, College of Osteopathic Medicine of the Pacific, Western University of Health Sciences, Pomona, CA 91766, USA

**Keywords:** Atherosclerosis, FOXO3, Hypercholesterolemia, Oxidative stress, SIRT3

## Abstract

Hypercholesterolemia is a major risk factor for atherosclerosis as oxidized-low-density lipoproteins (ox-LDL) contribute to the formation of foam cells and inflammation. Increased immune cell infiltration and oxidative stress induce instability of a plaque. Rupture of the unstable plaque precipitates adverse ischemic events. Since reactive oxygen species (ROS) play a critical role in plaque formation and vulnerability, regulating ROS generation may have therapeutic potential. Sirtuins, specifically sirtuin-3 (SIRT3), are antigenic molecules that can reduce oxidative stress by reducing mitochondrial ROS production through epigenetic modulation. Lack of SIRT3 expression is associated with dysregulation of ROS and endothelial function following high-fat high-cholesterol diet. SIRT3 deacetylates FOXO3a (Forkhead transcription factor O subfamily member 3a) and protects mitochondria against oxidative stress which can lead to even further protective anti-oxidizing properties. This study was designed to investigate the association between hyperlipidemia, intimal injury, chronic inflammation, and the expression of NAD-dependent deacetylase SIRT-3, FOXO3, antioxidant genes, and oxidative stress in carotid arteries of hypercholesterolemic Yucatan microswine. We found that intimal injury in hypercholesterolemic state led to increased expression of oxidative stress, inflammation, neointimal hyperplasia, and plaque size and vulnerability, while decreasing anti-oxidative regulatory genes and mediators. The findings suggest that targeting the SIRT3-FOXO3a-oxidative stress pathway will have therapeutic significance.

## 1. Introduction

Cardiovascular Diseases (CVDs) including coronary artery disease, strokes, and heart attacks are the leading cause of death in the world for men, women, and most if not all racial and ethnic subgroups in the United States [1]. It is estimated that 697,000 people died from heart disease alone in 2020, which is approximately around 32% of all deaths. CVDs, healthcare services, medications, and lost productivity cost the United States around $229 billion [2]. CVDs are a group of disorders affecting various components of the cardiac and vascular systems, significantly impacting the body’s hemodynamics, metabolism, immunity, cellular growth and repair, and responses from the nervous system [3]. CVD is a multifactorial disease and may arise from numerous etiologies such as ischemia due to atherosclerosis, arrhythmias such as atrial fibrillation, or viral infection leading to rheumatic heart disease, chronic inflammation, activation of inflammatory mediators, and secretion of damage-associated or pathogen-associated molecular proteins. However, the most common etiology leading to the pathophysiology of CVD remains to be atherosclerosis [4–15].

Atherosclerosis is defined as accumulation of fatty deposits and thickening of the intimal layer of arteries. The atheromatous plaques consist of fatty material, cellular debris, cholesterol, smooth muscle cells, leukocytes, and immune cells comprising the necrotic core [16, 17]. The pathogenesis of atherosclerosis, a chronic inflammatory condition, comprises of initial activation and dysfunction of endothelial cells, release of adhesion molecules, and recruitment of dendritic cells, macrophages, neutrophils, and lymphocytes. Lesions along the intima also attract smooth muscle cells migrating from the media layer [18]. Chronic inflammation induces the progression of the fibro-atheromatous plaque (stable) to vulnerable plaque (unstable) prone to rupture. Inflammatory mediators including triggering receptors expressed on myeloid cells (TREM)-1, toll-like receptors (TLRs), damage-associated molecular proteins (DAMPs), S100 proteins, pro-inflammatory cytokines, and immune cells including macrophages and dendritic cells play a critical role during the development of vulnerable plaque [4–15].

Oxidized low-density lipoproteins (oxLDL) which predominantly consist of cholesterol penetrate intima following the expression of pro-inflammatory cytokines [16]. Deficiency of LDL-receptor is a major cause for hypercholesteremia and can accelerate the destabilization of atheromatous plaques [19]. Increased expression of S100 proteins and receptors for advanced glycation end products (RAGE) due to oxLDL in the plaque may also increase plaque vulnerability which in turn increases the incidence of adverse cardiac events. Plaque vulnerability may also be increased due to increased oxidative stress causing endothelial dysfunction, hypoxic infarcts, and sustained inflammation [20, 21]. Reactive Oxygen Species (ROS), generated during hypoxia or injury regulating various cellular functions, induce oxidative stress and endothelial dysfunction, making the artery more prone to lesions [22]. Increased oxidative stress induces the formation of oxidized LDL from LDL, which is an important marker of atherosclerotic stress. Specifically, the presence of free radicals can oxidize and destabilize lipids and proteins in the cellular membrane [22].

Since ROS plays a critical role in plaque formation and vulnerability, regulating ROS generation may have therapeutic potential. In addition to lowering fat infiltration, Sirtuins, specifically SIRT3, are antigenic molecules that can reduce oxidative stress through epigenetic modulation. Lack of SIRT3 expression is associated with dysregulation of ROS and endothelial function following a high-cholesterol diet [23]. Localized to mitochondria, SIRT3 is found to protect mitochondrial DNA integrity, which is constantly under stress due to oxidative markers highly expressed in mitochondria. SIRT3 deacetylates FOXO3a (Forkhead transcription factor O subfamily member 3a), which can lead to even further protective anti-oxidizing properties [24]. Epigenetic regulation of the SIRT-FOXO3 axis can lead to an overall reduction of ROS and lower oxidative stress along with healing through the endothelial lesions that can help attenuate plaque progression.

Vascular smooth muscle cells (VSMCs) may also release mediators and proteases to promote further breakdown of the extracellular matrix [25]. However, it is thought that FOXO3a is activated and causes a cascade of pro-apoptotic targets. Since changes in the expression of FOXO3a can have a significant impact throughout the body, it is important to isolate protective mechanisms of the transcription factor that could lead to plaque attenuation. Since adverse ischemic events are triggered by plaque vulnerability, stabilizing atheromatous plaques is an important preventive measure and possible therapy to attenuate ischemic events in CVDs [26]. Reduction of plaque size and chronic inflammation in plaque could lead to attenuated plaque vulnerability and reduce the number of ischemic attacks and thrombotic events. In this study, we investigated the association between hyperlipidemia, intimal injury, the expression of NAD-dependent deacetylase sirtuin-3 (SIRT-3), FOXO3, and oxidative stress.

## 2. Methodology

### 2.1 Tissue collection and processing

This study used the collected carotid artery tissues from hyperlipidemic Yucatan microswine being used for another IACUC-approved (R19IACUC026) ongoing study in the Department of Translational Research at the Western University of Health Sciences at Pomona, CA. The tissues were processed using a tissue processor and 5μm thin sections were used in all experiments. The study groups included swine right ascending pharyngeal arteries with intimal injury with angioplasty and then treated with ox-LDL followed by vehicle control (30% ethanol) and with TLR4 inhibitor (TAK-242 dissolved in ethanol) [20]. Left-side internal carotid arteries without any intervention or treatment were included as biological controls. A total of 21 samples (7 samples in each group) were included in this study. We performed the power analysis with an α value of 0.05 and at least 90% power to detect about 30% change. The minimum number of swine for statistical analyses was 7 in each group.

### 2.2 Hematoxylin and Eosin and Movat-Pentachrome staining

Hematoxylin and eosin (H&E) staining was performed following the standard protocol in our lab. Briefly, after deparaffinization and rehydration of the sections through a series of xylene, alcohol, and distilled water, the tissue sections were stained with hematoxylin (45 seconds) followed by eosin (8-10 dips). The stained slides were mounted with xylene-based mounting media. Pentachrome staining was done using a modified pentachrome kit (Cat no. KTRMPPT from American MasterTech scientific laboratory supplies) following the manufacturer’s protocol and standard lab procedure. Stained tissue sections were scanned at 100μm using a light microscope (Leica DM6). Scanned images were evaluated for histomorphological analyses and blindly reviewed by two other independent observers for the presence and vulnerability of plaque and the presence and severity of inflammation.

### 2.3 Immunostaining

Immunohistochemistry was performed using the peroxidase anti-peroxidase method using a secondary antibody conjugated to horseradish peroxidase (HRP). The paraffin fixed sections were deparaffinized, rehydrated, and antigen retrieved using 1% citrate buffer (Sigma Aldrich # C9999) for 45 minutes before immunostaining as per the standard protocol in our laboratory. Briefly, the slides were washed with phosphate-buffered saline (PBS) after antigen retrieval and tissue section was encircled using a Pep Pen. The tissue sections were incubated with 3% hydrogen peroxide (Sigma Aldrich # H1009) for 15 minutes and washed with PBS for 5 minutes each three times. Blocking for nonspecific antigens was done using the blocking solution from the Vectastain Elite ABC kit (Vector Labs) and the tissue sections were incubated for 1 hour at room temperature. After tipping off the blocking solution, the tissue sections were incubated overnight with the primary antibodies HIF-1α (ab1), FOXO3a (NB100-614), AngIIT2R (ab9391), SIRT3 (MBS422614), HSP90 (ab13492) with a dilution factor of 1:50 to 1:200 after titration. The slides were washed 3 times for 5 minutes each with 1x PBS and then incubated with the secondary antibody (Vectastain Elite ABC kit) for 30 minutes at room temperature. The slides were rinsed 3 times with 1x PBS, followed by incubation with the Vectastain ABC horseradish peroxidase (HRP) for 30 minutes at room temperature. The tissue sections were then rinsed with 1x PBS followed by incubation with 3,3′-diaminobenzidine (DAB) (Thermo Scientific, Cat # 34002) for 2 to 5 minutes until the development of the brown color of the DAB. Tissue sections were washed with water once and then stained with hematoxylin for 20-30 seconds. The slides were rinsed in running tap water for 5 minutes and mounted with a xylene-based mounting medium. The stained slides were imaged with a Leica DM6 microscope at a scale of 100 μm. The high-magnification images from each tissue section were manually analyzed for average stained intensity and stained area using Fiji Image J. Three sections per sample and 3-5 images from each stained section for each swine were used for statistical analysis.

### 2.4 Real-Time Quantitative Polymerase Chain Reaction (RT-qPCR)

Total RNA was extracted using TRIZOL reagent (#93289, Sigma, St Louis, MO, USA) following manufacturer’s instructions, and RNA yield was measured using Nanodrop 2000. The cDNA was prepared using an iScript kit (BioRad, USA) following the manufacturer’s instructions. RT-qPCR was performed in triplicate using AzuraView GreenFast qPCR Blue Mix HR using the CFX96 RT-PCR system (BioRad Laboratories, Hercules, CA, USA). The primers were obtained from Integrated DNA Technologies (Coralville, IA, USA) (Table 1). The PCR cycling conditions were 5 min at 95°C for initial denaturation, 40 cycles of 30s each at 95°C (denaturation), 30 s at 55–60°C based on the primer annealing temperatures, and 30s at 72°C (extension) followed by melting curve analysis. Fold changes in mRNA expression relative to controls were analyzed using the 2-^^T method after normalization with 18S. Each experiment was repeated for three biological replicates (n = 3).

### 2.5 Statistical analysis

Data are presented as the mean ± SD (unless mentioned for mean ± SEM). Data was analyzed using GraphPad Prism 9. The comparison between the two groups was performed using a Student’s t-test and One-way ANOVA with Bonferroni’s post-hoc correction for more than two groups. A probability (p) value of <0.05 was accepted as statistically significant.

## 3. Results

**Figure 1:**
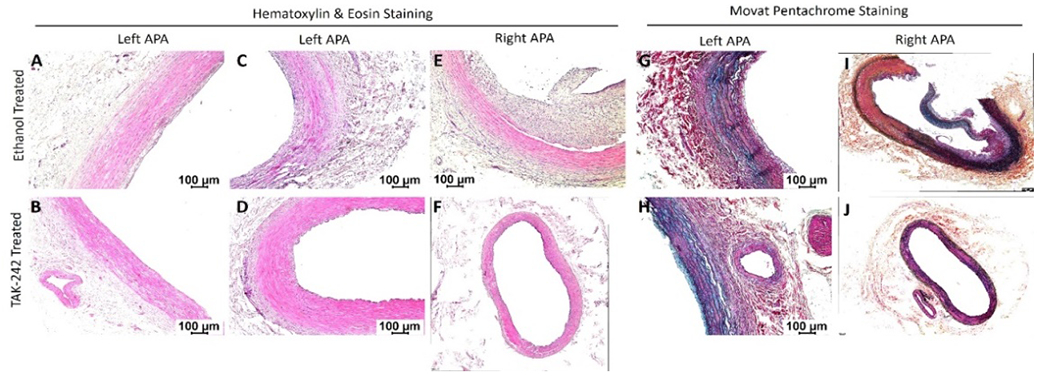
Hematoxylin and eosin staining of left and right ascending pharyngeal arteries (APA - equivalent to internal carotid arteries) in 30% ethanol (vehicle) and TAK-242 treated microswine. H&E staining in right and left APA in ethanol group (panels A, C, and E) and TAK-242 group (panels B, D, and F) arteries. Movat-Pentachrome staining in right and left APA ethanol group (panels G and I) and TAK-242-treated group (panels H and J) arteries. All images were scanned with a scale of 100μm. These images are representative images of all swine included in this study.

Hematoxylin and eosin and Movat-Pentachrome staining: H&E staining of the left ascending pharyngeal artery (APA equivalent to left internal carotid artery) without any intervention revealed no intimal hyperplasia in 6 out of 7 swine while a stable plaque with minimal occlusion was present in one swine (1 out of 7). Left APA revealed a lack of fibrosis around tunica adventitia (n = 2 out of 7) and mild fibrosis in tunica adventitia (n = 2 out of 7). The tunica media thickening was present in 2 out of 7 arteries and focal loss of tunica media in 1 out of 7. There is also minimal focal tunica media inflammation (n = 1 out of 7) and mild transmural inflammation (n = 1 out of 7) (Figure 1 panels A-D). Staining of the right APA in the ethanol group with angioplasty revealed stable plaque (n=2 out of 7), vulnerable plaque with hemorrhage (n = 1 out of 7), presence of necrotic core (n = 2 out of 7), and no plaque in two arteries. There was also focal intimal inflammation (n = 3 out of 7), mild transmural inflammation (n = 2 out of 7), and dense transmural inflammation (n = 2 out of 7). One artery showed thickened media (n = 1 out of 7). The right internal carotid artery (RIC) in the TAK-242-treated group with angioplasty showed stable plaque (n=1 out of 7) and presence of necrotic core (n = 1 out of 7). There was also thickening of the tunica media (n = 2 out of 7) and the tunica intima (n = 2 out of 7) in right APA in TAK-242-treated group. RIC with plaques showed mild focal intimal inflammation (n = 2 out of 7) and mild transmural inflammation (n = 1 out of 7). Thus, left APA showed minimal neointimal hyperplasia and lumen occlusion compared to right APA in ethanol and TAK-242 group which showed more fibrosis, medial thickening, and greater inflammation though these findings were more severe in the ethanol group compared to the TAK-242 group.

### 3.1 Real Time-PCR

**Figure 2:**
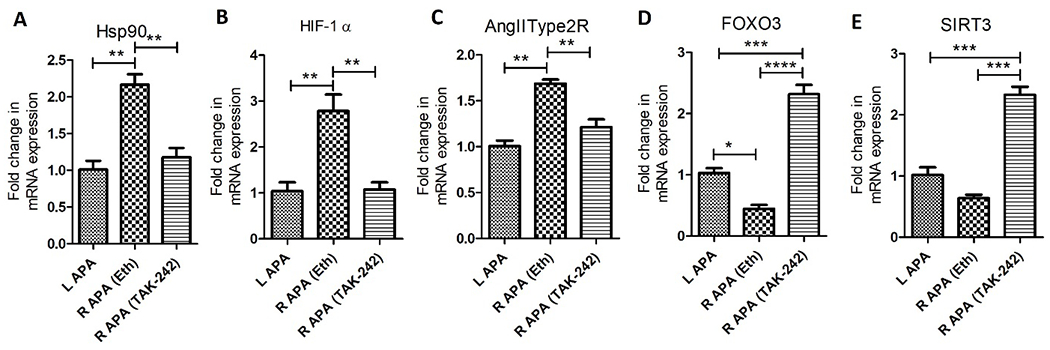
Expression of mRNA transcripts of heat shock protein (HSP)-90 (panel A), hypoxia-inducible factor (HIF)-1α (panel B), angiotensin II type 2 receptor (AngIIType2R; panel C), forkhead box O (FOXO)3 (panel D), and sirtuin-3 (SIRT3) (panel E) in right and left ascending pharyngeal arteries in TAK-242 and ethanol (Eth) treated swine. All data are presented as mean ± SD. **p<0.01 and ***p<0.001.

The findings from the RT-PCR revealed significantly increased fold change in mRNA expression of Hsp90 in right APA (ethanol) compared to left APA (p=0.003) and right APA TAK-242 (p=0.006) while there was no significant difference between left APA and right APA TAK-242 (p=0.389) (Figure 2 panel A). The fold change in the mRNA expression of HIF-1α was significantly increased in right APA (ethanol) compared to left APA (p=0.012) and right APA TAK-242 (p=0.012) while there was no significant difference between the left APA and right APA TAK-242 group (p=0.896) (Figure 2 panel B). The fold change in the mRNA expression of AngIIT2R was significantly increased in the right APA (ethanol) compared to left APA (p=0.008) and right APA TAK-242 group (p=0.008) while there was no significant difference between the left APA and right APA TAK-242 (p=0.123) (Figure 2 panel C). The fold change in the mRNA expression of FOXO3a was significantly decreased in the right APA (ethanol) compared to left APA (p=0.004) and right APA TAK-242 (p=0.001) and the fold change in mRNA expression in right APA (ethanol) was significantly decreased compared to right APA TAK-242 (p=0.0017) (Figure 2 panel D). The fold change in mRNA expression of SIRT3 was decreased in the right APA (ethanol group) compared to left APA (p=0.051) and significantly decreased compared to right APA in TAK-242-treated group (p=0.0003) and the fold change in mRNA expression in right APA (ethanol) was significantly decreased compared to right APA in TAK-242-treated group (p=0.0019) (Figure 2 panel E).

### 3.2. Immunostaining

**Figure 3:**
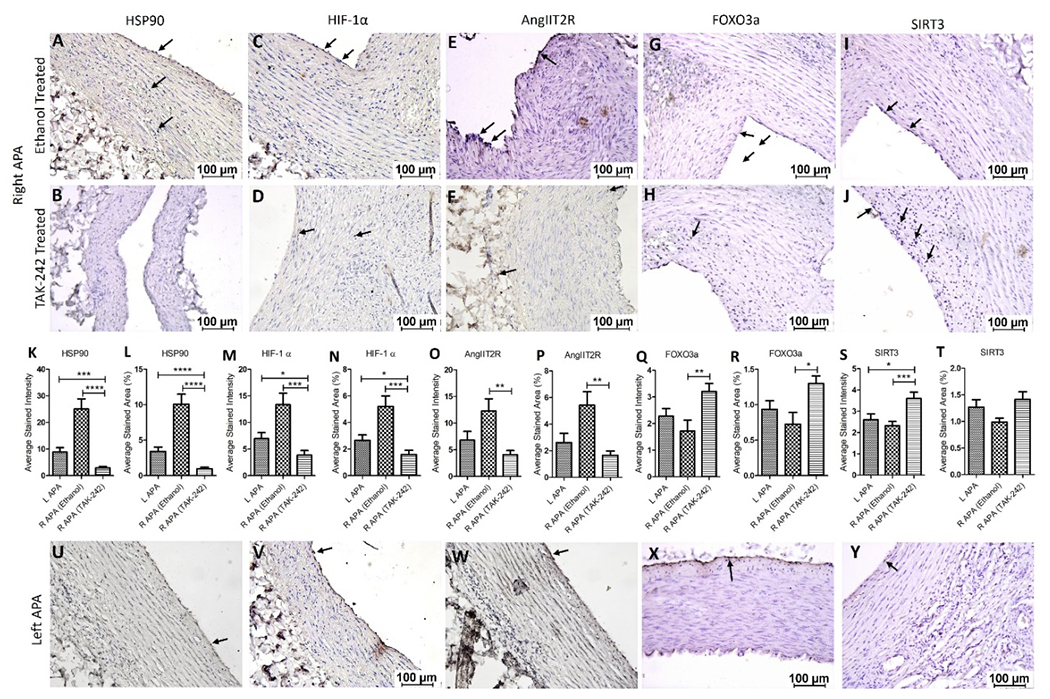
Immunohistochemistry for heat shock protein (HSP)-90, hypoxia-inducible factor (HIF)-1α, angiotensin II type 2 receptor (AngIIT2R), forkhead box O (FOXO)3, and sirtuin (SIRT)3 in right ascending pharyngeal arteries (APA) treated with ethanol alone (vehicle) or TAK-242 dissolved in ethanol in the hypercholesterolemic Yucatan microswine. Right APAs (panels A-J), left APA s (panels U-Y), and average stained intensity and average stained area are shown in percent (panels K-T). All images were scanned with a scale of 100μm. These images are representative images of all swine included in this study. All data are presented as mean ± SEM. *p<0.05, **p<0.01, ***p<0.001 and ***p<0.001.

Immunohistochemistry of the collected tissues showed immunopositivity for HSP90, HIF-1α, AngIIT2R, FOXO3a, and SIRT3. The immunopositivity for HSP90 was significantly increased in the right APA ethanol group (p=0.0124) and decreased in right APA TAK-242 group (p=0.0019) compared to the left APA. The immunopositivity for HSP90 was significantly increased in right APA ethanol compared to right APA TAK-242 (p=0.0003) (Figure 3 panels A, B, and U). The immunopositivity for HIF-1α was significantly increased in right APA ethanol (p=0.0211) and significantly decreased in right APA TAK-242 group (p=0.038) compared to left APA. The immunopositivity for HIF-1α was significantly increased in right APA ethanol group compared to right APA TAK-242 group (p=0.0008) (Figure 3 panels C, D, and V). The immunopositivity for AngIIT2R was increased in the right APA ethanol (p=0.1016) and decreased in the right APA TAK-242 group (p=0.1006) compared to the left APA. The immunopositivity for AngIIT2R was significantly increased in the right APA ethanol group compared to right APA TAK-242 group (p=0.0013) (Figure 3 panels E, F, and W). The immunopositivity for FOXO3a was decreased in the right APA ethanol group (p=0.253) and significantly increased in right APA TAK-242 group (p=0.0306) compared to the left APA. The immunopositivity for FOXO3a was significantly decreased in right APA ethanol group compared to right APA TAK-242 group (p=0.005) (Figure 3 panels G, H, and X). The immunopositivity for SIRT3 was decreased in the right APA ethanol group (p=0.404) and significantly increased in right APA TAK-242 group (p=0.0196) compared to the left APA. The immunopositivity for SIRT3 was significantly decreased in the right APA ethanol group compared to the right APA TAK-242 group (p=0.0012) (Figure 3 panels I, J, and Y). These findings were collaborated with the results of average stained intensity and percent area (Figure 3 panels K-T).

## 4. Discussion

Histological staining revealed normal APA on the left side in both ethanol and TAK-242 groups supporting the findings of a previous study with normal left APAs in swine where right APA was treated with oxLDL [20]. Mild fibrosis in tunica adventitia that was observed in two left APAs is most likely a response to sustained elevation of shear stress since some of the areas in the carotid arteries experience highest wall shear stress in the vascular system, or due to systemic response of the intervention on the right side carotid artery [27]. The inflammation accompanies the areas of neointimal hyperplasia as observed in one sample of the left APA and this might be due to the systemic inflammatory response after injury on the right side or due to hyperlipidemic diet or shear stress [28]. The pathology in the right APA of the ethanol group may be due to intimal injury and increased inflammation due to angioplasty synergizes with the presence of hyperlipidemia [29].

TAK-242 inhibits TLR-4 causing downregulation of many downstream inflammatory cascades involving NF-κB [30]. TLR-4 inhibition results in reduced secretion of cytokines and recruitment of lymphocytes to the site of injury [31]. Thus, attenuated inflammation in the right APA treated with TAK-242 may be the result of attenuated TLR-4 by TAK-242. TLR-4 inhibition also significantly reduces plaque inflammation and size [32] and smaller plaque or no plaque or only minimal intimal thickening in right APA TAK-242 arteries compared to right APA in ethanol-treated arteries suggests the beneficial therapeutic effect of TAK-242 [31]. Furthermore, there was an increased presence of inflammation and thickening of both tunica intima and media layers in the right APA TAK-242-treated group in comparison to the left APA despite the anti-inflammatory treatment. This is most likely due to hyperlipidemia and the expression of S100 proteins and inflammatory mediators such as interleukins and TNF-α following injury to the tunica intima after angioplasty [15, 20, 33].

Histological characterization of the arteries suggests that intimal injury with angioplasty in a hyperlipidemic environment induces plaque formation. The intimal injury also results in the activation of the renin-angiotensin-aldosterone (RAAS) system locally, which is an essential homeostatic pathway that maintains plasma sodium, arterial blood pressure, and extracellular volume within normal limits for the body’s proper functioning [34, 35]. Triggered by either the activation of the sympathetic autonomic nervous system or decreased filtration of sodium in the nephrons of the kidneys, renin is secreted to cause a cascade activating Angiotensin II (AngII). Blocking AngII has been found to have decreased damage to brain and cardiac tissues [36]. Activation of AngII promotes macrophage recruitment and mobility, dendritic cell maturation, antigen uptake/processing, proliferation of expression of TLR-4, ROS levels, apoptosis, and chemokine production [37]. AngII promotes endothelial cell dysfunction through COX2 activation and production of COX2 and MCP-1 [38]. AngII blockade reduces vessel inflammation and severely attenuates macrophage infiltration. AngII is pro-inflammatory by binding to its receptors AngII Type 1 Receptor and Ang II Type II Receptor (AngIIType2R) by mediating free radical production that promotes mitochondrial dysfunction [39]. While the activation of AngII Type 1 receptor inhibits cellular differentiation and promotes proliferation, the type 2 receptor inhibits proliferation and inhibits differentiation. AngIIType2R activation specifically also mediates vasodilation and NO production. AngIIType2R is highly expressed in fetal tissue but rapidly declines after the neonatal period, highest specifically in the endothelium of coronary arteries [40, 41]. AngIIType2R is also increased in pathological conditions such as vascular injuries, explaining the significant increase in AngIIType2R in the right APA (both ethanol and TAK-242 groups) compared to the left APA [42]. AngIIType2R is also known to be negatively regulated by various factors such as TGF-β, AngII, norepinephrine, and insulin-like growth factors [38]. TLR4 is also known to have a pivotal role in the expression of AngII, as the lack of TLR4 in TLR4lps-d mice inhibits AngII-induced oxidase activity [43]. Inhibition of TLR4 expression through TLR4-binding inhibitors reduces the expression of AngII and an increased expression of AngIIType2R in both RT-PCR and Immunohistochemistry and this may be due to lack of AngII levels negatively regulating AngIIType2R [44]. Further, since intimal injury-induced RAAS activation is associated with inflammation and oxidative stress in the pathogenesis of plaque formation and atherosclerosis [45], we examined the arteries for the presence of oxidative stress.

The Hsp90 (Heat-shock proteins) family of proteins is a cluster of highly conserved molecules involved with many roles such as protein translation in cellular regulation, homeostasis, and apoptosis. A study done by Chatterjee and collaborators showed that inhibition of Hsp90 production is correlated with the attenuation of inflammatory parameters in locally induced areas of inflammation as well as systematically [46]. Hsp90 inhibitors which have been mainly used to reduce tumor growth have demonstrated a reduction in inflammation-caused oxidative stress [47]. Hsp90 stands to be an important marker of cellular damage caused by necrotic intima in atherosclerosis. Reducing the levels of Hsp90 has also been shown to decrease MCP-1, NF-kB expression, and vascular smooth muscle cell migration, all of which are prognostic factors of atherosclerosis [47]. Although Hsp90 is shown to reduce NF-kB levels, there seems to be a dynamic relationship between both as activated NF-kB induces Hsp90B expression [48]. Hsp90 gene expression is also upregulated by other factors such as IL-6, STAT3 (signal transducer and activator of transcription 3), and IFN-γ (interferon-γ). Inhibition of TLR4 in microglia cells is strongly correlated with the dramatic reduction of IFN-γ [49]. Thus, simultaneous reduction of both activated NF-kB and IFN-γ could prompt a reduction of Hsp90 factors as there was a significant reduction in Hsp90 expression in the right APA TAK-242 groups compared to the left APA. However, an increased HSP90 in the right APA (both ethanol and TAK-242 groups) compared to the left APA might be due to intimal injury and the presence of hyperlipidemia. Vascular injury resulting from angioplasty-induced injuries can mimic the environment induced by ischemic and hypoxic conditions [50]. Thus, we can predict a reciprocal relationship between TLR4 inhibition and increased Hsp90 levels as there was a significant increase in Hsp90 expression in the TAK-242 group compared to the control group when both groups induced intimal injury using angioplasty.

Hypoxia-inducible factor (HIF-1) is a transcription activator of various protein factors such as PHD, pVHL, ARD-1, and p300/CBP that is oxygen-dependent [51]. In normoxic conditions, HIF-1α is degraded by the von Hippel-Lindau tumor suppressor gene product (pVHL)- mediated ubiquitin-proteasome pathway. In low oxygen conditions, HIF-1α is stabilized and activates target genes related to angiogenesis, cellular proliferation, glucose, and iron metabolism. Due to its involvement with tumor genesis, it is frequently targeted by anti-cancer therapies as blocking HIF-1α can inhibit tumor growth. HIF-1α is used frequently as a marker of oxidative stress and hypoxia [52]. HIF-1α is thought to have a protective effect in vascular disorders as it induces mature vessel formation. Since angioplasty-induced intimal injuries mimic other endothelial injuries in downstream activation of inflammatory cascades, HIF-1α is expected to increase in the presence of injuries to signal for angiogenesis and cellular repair. Although HIF-1α is strongly correlated with inflammation, oxygen-dependent activation is primarily sufficient to increase HIF-1α levels [53]. Henceforth, we can explain the significant increase of HIF-1α in the presence of intimal injury in the right APAs of the ethanol group compared to the right APAs of the TAK-242 group. An increased HIF-1α in right APA compared to left APA may be due to inhibition of inflammatory response and decreased intimal hyperplasia due to local TAK-242 treatment while an increase in left APA due to systemic inflammation.

The Forkhead Box (FOX) family of transcription factors play critical roles in development, metabolism, and aging, specifically implicated in inflammation and tumor pathogenesis [54]. Specifically, FOXO3 or Fkhrl1 is a mammalian homolog of the longevity gene DAF-16 from Caenorhabditis elegans. FOXO3 is understood to be generally anti-inflammatory as its deficiency leads to spontaneous lymphoproliferation, T Cell hyperactivation, inflammatory myopathies, and idiopathic autoimmune disorders [55]. Molecularly, its deficiency also leads to NF-kB hyperactivation and thus overexpression of IL-2 and IFN-γ. In addition, FOXO3 appears to produce pro-apoptotic effects in CD4+ T Cells and macrophages possibly through the Egr1-PTEN pathway as observed in the breakdown of lymph node microenvironment in HIV patients. FOXO3 contributes to survival in chronic inflammation, as its dephosphorylation and subsequent activation is through mitogen-dependent stimulation via the PI3-Akt pathway found most often in chronic inflammatory states [56]. Due to its protective and anti-inflammatory effects, FOXO3 can remain a barrier to the attainment of complete activation of inflammatory cytokines [57]. In a study investigating the phagocytic capacity of microglia cells, autophagy-related genes were found to be significantly suppressed due to TLR4 activation while active FOXO3 induced autophagy. Macrophages with reduced autophagic capacity with present TLR4 activation were found in cells with FOXO3 downregulation, indicating a TLR4-induced inhibitory effect on FOXO3. With the treatment of TAK242, there would be an increase in the expression of FOXO3 in the states of inflammation. An increased expression of FOXO3 in right APA TAK 242 in comparison to both left APA and right APA ethanol groups and a decreased FOXO3a in R APA ethanol group are in line with previous findings that attenuation of inflammation is associated with FOXO3a upregulation and vice-versa.

Sirtuins are a family of nicotinamide adenine dinucleotide–dependent deacetylases (SIRT1–7) that are involved in the delay of aging through protective effects in vascular endothelial dysfunction, metabolic syndrome, cardiomyopathy, and tumor growth [58]. Sirtuin level overall decreases with aging as well as in conditions such as obesity, sedentary lifestyle, and stress, all of which are known risk factors for CVD. Studies investigating mice lacking SIRT3 have been shown to have decreased levels of fatty acid oxidation compared to wild-type mice, possibly an important factor in high LDL levels that help progress to atherosclerosis [59]. SIRT3 regulates mitochondrial and biosynthetic functions such as glucose and fatty acid metabolism [60]. SIRT3 is also shown to be specifically cardioprotective by maintaining mitochondrial ATP levels, reducing oxidative stress-mediated cellular damage, and reducing hypertrophy. This effect is thought to be the same systematically as well as loss of function of SIRT3 has been associated with increased hypertrophy, fibrosis, obesity, insulin resistance, and serum lipid levels. This can be due to the hyperacetylation of severe mitochondrial proteins such as long-chain acyl-coenzyme A dehydrogenase (LCAD), inactivating fat breakdown [58]. SIRT3 can also activate isocitrate dehydrogenase (IDH2), SOD2, and CAT, all key enzymes in reducing the cellular burden of ROS [61]. This suggests that decreased levels of SIRT3 are associated with increased oxidative stress and our findings of attenuated SIRT3 with increased expression of HSP90, HIF-1α, and AngIIT2R in the right APA of ethanol group support the notion that decreased SIRT3 may contribute to plaque vulnerability. Further, attenuation of inflammation by inhibiting TLR-4 with TAK-242 stabilizes the plaques or attenuated the formation of plaques and its association with increased SIRT3 in the right APA TAK-242 group suggest the beneficial effect of SIRT3. This suggests that SIRT3-FOXO3a-oxidative stress plays a critical role in plaque vulnerability and may be a therapeutic target to attenuate plaque vulnerability [62].

In summary, the results of this study suggest that intimal injury in the hyperlipidemic state increases oxidative stress, inflammation, neointimal hyperplasia, and plaque size and vulnerability but decreases anti-oxidative regulatory genes. Markers of inflammation and oxidative stress such as Hsp90, HIF-α, and AngIIT2R were increased in tissues that had undergone angioplasty-induced intimal injury and were subsequently reduced in tissues that had undergone TLR-4 inhibitory, anti-inflammatory treatment. FOXO3, which is a transcription factor responsible for the generation of pro-apoptotic, anti-inflammatory pathways, was increased in tissues treated with TAK-242. Future studies to investigate the therapeutic role of targeting SIRT3-FOXO3a-oxidative stress are warranted.

## 5. Conclusion

The results suggest that intimal injury increases oxidative stress, inflammation, and plaque formation and decreases anti-oxidative mechanisms while attenuating inflammation has a beneficial effect. There is an association between intimal injury, SIRT3, and FOXO3a expression. Thus, attenuating plaque vulnerability through reducing oxidative stress inflammatory cascades by targeting SIRT3 is an important therapeutic strategy. However, a clear mechanism of how SIRT3 leads to phosphorylation and activation cascade of FOXO3, how these pathways reduce inflammatory chemokines, and what is the effect of attenuating inflammation on this pathway are still very much to be investigated. Further, epigenetic regulation of intimal injury-SIRT3-FOXO3a-oxidative stress axis in the presence of intimal injury and hypercholesterolemia warrants further research.

## Figures and Tables

**Table 1:** Nucleotide sequence of genes used in RT-PCR in this study.

Gene	Forward sequence (5’--3’)	Reverse sequence (5’--3’)
HSP90	CTGCTTCCGGCAGTTCTT	CATACTCCTTCATGGCCTTCTC
HIF-1α	CCACCTCTGGACGTGCTTTT	CTTCCATGGCGAATCTGTGC
AngIIT2R	CCTCTCTGGGCAACCTATTATTC	ACATGTTCAGGGTCAGGAAAG
SIRT3	CTACTACAGCACCCTCCAAAG	GGAAGTAGTGAGCAGTGTTAGG
FOXO3a	ACAAACGGCTCACTCTGTCC	GTTGCTGTCGCCCTTATCCT

## Data Availability

Data are available upon request from the authors through proper channels.

## Limitation of the study and Future Directions

Although we used 7 swine in each experimental group, the findings could be supported with additional sample size to get more consistent results. Further, the data should be supported by in-vitro studies investigating the relationship between SIRT3-FOXO3a-oxidative stress in the presence and absence of inflammatory cytokines and hyperlipidemia. The study may also be supported by in-vitro and in-vivo investigations for epigenetic factors and their effects in various groups.
